# CKG-TPI: integrating collaborative knowledge graph with sequence interactions for TCR–peptide binding specificity

**DOI:** 10.1093/bib/bbaf486

**Published:** 2025-09-22

**Authors:** Yue Liu, Haoyan Wang, Guohua Wang, Yadong Liu, Tao Jiang, Yadong Wang

**Affiliations:** Faculty of Computing, Harbin Institute of Technology, Harbin, Heilongjiang 150001, China; Faculty of Computing, Harbin Institute of Technology, Harbin, Heilongjiang 150001, China; Faculty of Computing, Harbin Institute of Technology, Harbin, Heilongjiang 150001, China; Faculty of Computing, Harbin Institute of Technology, Harbin, Heilongjiang 150001, China; Zhengzhou Research Institute, Harbin Institute of Technology, Zhengzhou, Henan 450000, China; Faculty of Computing, Harbin Institute of Technology, Harbin, Heilongjiang 150001, China; Zhengzhou Research Institute, Harbin Institute of Technology, Zhengzhou, Henan 450000, China; Faculty of Computing, Harbin Institute of Technology, Harbin, Heilongjiang 150001, China; Zhengzhou Research Institute, Harbin Institute of Technology, Zhengzhou, Henan 450000, China

**Keywords:** T-cell receptor (TCR), peptide, TCR–peptide binding, collaborative knowledge graph, graph neural network

## Abstract

Accurately identifying interactions between T-cell receptors (TCRs) and peptides is a fundamental challenge in immunology, with significant implications for vaccine design and immunotherapy. While computational methods offer efficient alternatives to labor-intensive experimental screening, achieving robust and accurate TCR–peptide binding prediction remains a challenging task. To address this, we propose collaborative knowledge graph (CKG-TPI), a novel prediction framework based on graph neural networks that integrates both interaction patterns between TCR and peptide sequences and their higher-order biological context through a constructed collaborative knowledge graph. Experimental results on multiple publicly available independent datasets demonstrate that CKG-TPI consistently outperforms state-of-the-art models. Specifically, it achieves a 9.89% improvement in area under the ROC curve compared to the strongest baseline model UnifyImmun, and a 23.93% increase in area under the precision-recall curve over the leading baseline method. Moreover, attention weight visualization and peptide-specific TCR screening validate the model’s effectiveness, underscoring its potential as a powerful tool for immunological research and therapeutic discovery.

## Introduction

Interactions between T-cell receptors (TCRs) and peptides is a core mechanism of the immune system for recognizing and responding to antigens, with significant applications in cancer immunotherapy, infectious disease prevention, and autoimmune disease research [1]. This T-cell-mediated immune response enables the monitoring of major histocompatibility complex-bound ligands presented on nearly all nucleated cells [2, 3]. The specificity of each TCR depends on its binding affinity to a particular antigenic peptide, and this binding interaction is vital in the immune response [4, 5]. Given the vast diversity of TCRs and peptides, determining specific TCR–peptide pairs remains a challenging and resource-intensive task. Identifying TCR–peptide binding interactions typically involves experimental methods such as high-throughput peptide library synthesis, TCR library construction, and high-throughput screening. These methods are both costly and time-consuming [6, 7], posting a major bottleneck in immunological research and the development of personalized immunotherapies.

With the continuous advancement of deep learning-based methods to model complex patterns and process large-scale data, numerous computational methods have been proposed to predict TCR–peptide binding specificity. For instance, ERGO [8] leverages natural language processing (NLP) techniques to encode TCR β-chain and peptide sequences to predict TCR–peptide binding. NetTCR-2.0 [9] utilizes convolutional neural networks (CNNs) to incorporate both TCR α- and β-chain information for improving binding prediction. MIX-TPI [10] further refines CNN-based feature extraction by integrating both amino acid sequences and physicochemical properties. TEIM [11] applies a few-shot learning strategy to model residue-level interactions based on limited sequence-level data, enhancing generalization. PanPep [12] adopts a meta-learning-based framework augmented with a neural Turing machine to address binding prediction for rare or previously unseen peptides. And UnifyImmun [13] employs cross-attention transformer architecture to simultaneously predict the bindings of peptides to both human leukocyte antigen (HLA) and TCR. Additionally, TCR-BERT [14] draws inspiration from NLP to employ unsupervised pretraining on large collections of unlabeled sequences to learn context-aware representations relevant to biological sequence modeling and immune recognition. Protein language models (PLMs) have demonstrated promising performance and strong generalization capacity. However, most PLMs are limited by their reliance on sequence data alone, neglecting the broader biological context of TCR–peptide interactions. Specifically, these models often overlook valuable auxiliary information such as functional annotations, genomic context, and ontological annotations. Consequently, their prediction performance remains suboptimal, which limits their practical application in immunology and precision medicine.

To address these limitations, we propose CKG-TPI, a hybrid model integrating higher-order biological context through a Collaborative Knowledge Graph (CKG) with sequence-level interaction features in an end-to-end manner. To our knowledge, CKG-TPI is the first attempt to systematically leverage heterogeneous biological entities and their intrinsic relationships for TCR–peptide binding prediction. The CKG-TPI framework models both direct and high-order associations between TCRs, peptides, and other biologically relevant entities. To effectively leverage the structure of the CKG, we introduce two core mechanisms: (i) Hierarchical neighbor propagation: the embedding of a node (TCR, peptide, or auxiliary feature) is iteratively updated by the embeddings of its neighboring nodes, allowing the model to capture rich and high-order dependencies within the knowledge graph. (ii) Dynamic importance aggregation [15, 16]: the neural attention mechanism dynamically learns the importance of neighbors during information propagation, allowing the attention weights to reveal the importance of high-order interactions during cascaded propagation.

Following the evaluation metrics established in previously published studies [17–21], our comprehensive evaluation across multiple independent datasets demonstrates that CKG-TPI outperforms state-of-the-art models in key metrics, including area under the ROC curve (AUC), area under the precision-recall curve (AUPR), accuracy (ACC), F1 score (F1), and Matthews correlation coefficient (MCC). These results confirm the effectiveness of incorporating structured biological knowledge into the predictive framework. The proposed model demonstrates not only improved predictive accuracy and robustness but also enhanced generalization across datasets. These findings highlight CKG-TPI as a valuable tool for immunological research and therapeutic development, including applications in neoantigen screening, autoimmune disease research, and personalized cancer immunotherapy.

## Materials and methods

### Overview of the CKG-TPI framework

**Figure 1:**
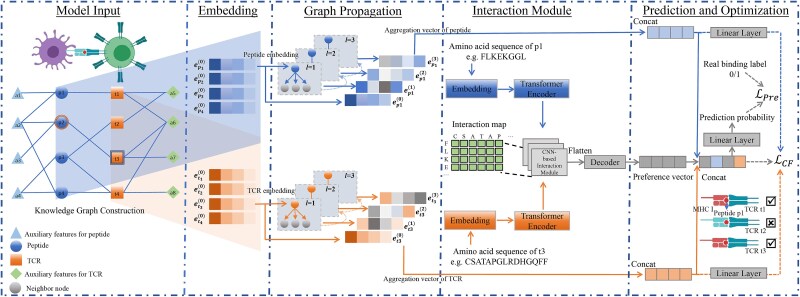
Overview of the proposed CKG-TPI framework for TCR-peptide binding prediction.

The objective of this study is to utilize the constructed CKG as input to predict the likelihood of TCR–peptide binding. We propose the CKG-TPI model, a novel end-to-end graph neural network framework that effectively captures high-order relationships among TCRs, peptides, and auxiliary biological features. Inspired by recommendation system [22], the framework of CKG-TPI consists of four major components (Fig. 1). (i) Embedding layer that encodes each TCR or peptide node as a vector by preserving the structure of CKG. (ii) Graph attention propagation module that recursively propagates node embeddings by aggregating information from their multi-hop neighbors. During each propagation, a graph attention network (GAT) is employed to dynamically learn the weight of each neighboring node. (iii) Sequence-aware interaction preference module which captures the sequence-specific representations of TCR and peptides using Transformer and extracts local and global interaction patterns from a pairwise interaction map using CNN. These features are fused to produce a comprehensive interaction preference vector for each TCR–peptide pair. (iv) Prediction module that aggregates multi-layer graph representations of TCRs and peptides with the preference vector from the sequence-aware module. This fusion yields a binding specificity score for each candidate TCR–peptide pair.

### Construction of collaborative knowledge graph

Prior to detailing the CKG-TPI model, we first explain the concept of CKG, the high-order connectivity between nodes, including TCRs, peptides, and rich auxiliary biological context in the graph. First, we represent known TCR–peptide pairs as a bipartite graph $G1$, defined as $G1=\{(p, pt,t\right)\ |\ p\in P,t\in T)\}$, where $P$ and $T$ represent the sets of peptide and TCR nodes, respectively. Here, $pt=1$ indicates a known interaction between peptide $p$ and TCR$t$, otherwise $pt=0$. We also incorporate auxiliary biological features for both peptides and TCRs, such as coding genes and functional annotations. The auxiliary information is organized into a knowledge graph $G2$ as a collection of subject-attribute-object triples [23]: $G2=\left\{\left(h,r, ta\right)|h\in P\cup T, ta\in A,r\in R\right\}$, where, each triple $\left(h,r, ta\right)$ describes a relation $r$ from the head node $h$ to the tail node $ta$. Here, $A$ denotes the nodes of auxiliary features, and $R$ means the set of relation types linking features to peptides or TCRs. For example, the CDR3β chain CASSDAGGGMAEAFF ($h$) may be linked to TRBJ1-1:01 ($ta$) and TRBV2-01 ($ta$) via encoding relations. The specific examples of the auxiliary features embedded in the constructed CKG are illustrated in Supplementary Figure S4. Based on this, we define the concept of CKG, which encodes the relationship and knowledge of peptides and TCRs into a unified relational graph. Each peptide is first represented as a triplet $\left(p, Interact,t\right)$, where $pt=1$ means an additional relationship $Interact$ between peptide $p$ and TCR $t$. Based on the peptides or TCRs and corresponding auxiliary features, the graph $G1$ can be integrated with $G2$ into a unified graph $G=\left\{\left(h,r, ta\right)|h, ta\in{E}^{\prime },r\in{R}^{\prime}\right\}$, where ${E}^{\prime }=P\cup T\cup A$ is the complete node set, and ${R}^{\prime }=R\cup \left\{ Interact\right\}$ extends the relation set with an explicit interact relation denoting known TCR–peptide binding.

### Construction of the embedding layer

To encode nodes and relationships into vector representations, we employ TransR [24] for knowledge graph embedding while preserving the graph structure. Specifically, if there exists a triple $\left(h,r, ta\right)$ in the graph, the embeddings can be learned by optimizing the translation principle ${e}_h^r+{e}_r\approx{e}_{ta}^r$. Here, ${e}_h,{e}_{ta}\in{R}^d$ and ${e}_r\in{R}^k$ represent the embeddings of $h$, $ta$, and $r$, respectively. ${e}_h^r\ \mathrm{and}\ {e}_{ta}^r$ represent the projections of ${e}_h$ and ${e}_{ta}$ in the relation $r$’s space. Therefore, the plausibility score of a given triple $\left(h,r, ta\right)$ is represented as:


(1) \begin{equation*} g\left(h,r, ta\right)={\left|\left|{W}_r{e}_h+{e}_r-{W}_r{e}_{ta}\right|\right|}_2^2 \end{equation*}

where ${W}_r\in{R}^{k\times d}$ is the transformation matrix for relation $r$, projecting entities from the $d$-dimensional entity space into the $k$-dimensional relation space. A lower score for $g\left(h,r, ta\right)$ indicates a more likely true triple, while a higher score suggests an invalid triple. To differentiate between valid and invalid triples, we optimize the pairwise ranking loss:


(2) \begin{equation*} {\displaystyle \begin{array}{c}{\mathcal{L}}_{KG}=\sum_{\left(h,r, ta,{ta}^{\prime}\right)\in T}-\mathit{\ln}\sigma \left(g\left(h,r,{ta}^{\prime}\right)-g\left(h,r, ta\right)\right)\end{array}} \end{equation*}

where $T=\left\{\left(h,r, ta,{ta}^{{\prime}}\right)\ |\ \left(h,r, ta\right)\in G,\left(h,r,{ta}^{{\prime}}\right)\notin G\right\}$, $\left(h,r,{ta}^{{\prime}}\right)$ is an invalid triple created by randomly replacing an entity from a valid triple, and $\sigma\ \left(\cdotp \right)$ represents the sigmoid function. During each epoch, this layer is first trained to learn the representations of knowledge graph nodes. It leverages a knowledge graph loss to enhance the model’s ability to represent nodes within the knowledge graph while acting as a regularizer to prevent overfitting.

### Construction of the graph attention propagation module

To enhance the model’s ability to capture the patterns of TCR–peptide binding, we incorporate biological context information, and further design a graph attention propagation module that recursively propagates embeddings along high-order connections in the knowledge graph. This module integrates GAT [25] to dynamically assign importance weights for cascaded propagation. For each layer, there are three components: information propagation, biological context-aware attention mechanism, and information aggregation. It is then extended to multiple layers. The advantage of this module is that it uses first-order connections to correlate representations of peptides, TCRs, and related auxiliary biological nodes.

For information propagation, the model propagates information recursively through the knowledge graph structure, allowing high-order relationships to be captured. An element can appear in multiple triples within the graph, acting as a bridge between them to propagate information. For example, consider the following propagation paths: ${a}_6\overset{r_2}{\to }{t}_3\overset{-{r}_1}{\to }{p}_2$ and ${a}_7\overset{r_3}{\to }{t}_3\overset{-{r}_1}{\to }{p}_2$. TCR ${t}_3$ takes features ${a}_6$ and ${a}_7$ as inputs to enrich its own representation, subsequently contributes to the representation of peptide ${p}_2$. Such feature propagation enables the transfer of information from auxiliary features to its neighbors. For an entity $h$, we define the ego-network [26] as the set of triples with $h$ as the head node ${N}_h=\left\{\left(h,r, ta\right)\mid \left(h,r, ta\right)\in G\right\}$. To capture the first-order connectivity structure of $h$, we compute a linear combination of its ego-network:


(3) \begin{equation*} {\displaystyle \begin{array}{c}{e}_{N_h}=\sum_{\left(h,r, ta\right)\in{N}_h}\pi \left(h,r, ta\right){e}_{ta}\end{array}} \end{equation*}

where $\pi \left(h,r, ta\right)$ is a propagation decay factor controlling how much information is transferred along the edge $\left(h,r, ta\right)$, indicating information flow from $ta$ to $h$ under relation $r$.

For biological context-aware attention mechanism, we introduce a relational attention mechanism to compute and update the importance weight $\pi \left(h,r, ta\right)$ as follows:


(4) \begin{equation*} {\displaystyle \begin{array}{c}\pi \left(h,r, ta\right)={\left({W}_r{e}_{ta}\right)}^T\mathit{\tanh}\left(\left({W}_r{e}_h+{e}_r\right)\right)\end{array}} \end{equation*}

where $\mathit{\tanh}$ [25] is the nonlinear activation function, ensuring attention scores are distance-dependent between ${e}_h$ and ${e}_{ta}$ in the relational space $r$. Next, we normalize the coefficients across all triples connected to $h$ by a softmax function:


(5) \begin{equation*} {\displaystyle \begin{array}{c}\pi \left(h,r, ta\right)=\frac{\mathit{\exp}\left(\pi \left(h,r, ta\right)\right)}{\sum_{\left(h,{r}^{\prime },{ta}^{\prime}\right)\in{N}_h}\mathit{\exp}\left(\pi \left(h,{r}^{\prime },t{a}^{\prime}\right)\right)}\end{array}} \end{equation*}

This mechanism ensures which neighboring nodes should be given more attention to capture collaborative signals. During forward propagation, the attention mechanism dynamically highlights the most informative parts of the data. Unlike information propagation in GCN [27] and GraphSage [28], our model not only leverages graph neighborhood structure, but also specifies the neighbors’ importance. Furthermore, unlike GAT, which uses only node representations as input, we model the relationship ${e}_r$ to encode additional information during propagation.

In information aggregation, we aggregate the information from element $h$ and its ego-network ${e}_{N_h}$ to obtain its updated representation: ${e}_h^{(1)}=f\left({e}_h,{e}_{N_h}\right)$. We use a Bi-Interaction Aggregator to implement the aggregation function $f\left(\cdotp \right)$ [22], which considers two types of feature interactions:


(6) \begin{equation*} {f}_{Bi- Interaction}= LeakyReLU\left({W}_{1\left({e}_h+{e}_{N_h}\right)}\right)+ LeakyReLU\left({W}_{2\left({e}_h\odot\,{e}_{N_h}\right)}\right) \end{equation*}

where $LeakyReLU$ [29] is the activation function, and ${W}_1,{W}_2\in{R}^{d^{\prime}\times d}$ are trainable weight matrices for extracting useful propagation information. ${d}^{\prime }$ is the transformation dimension, and $\odot$ denotes element-wise multiplication.

Furthermore, we extend the information propagation and aggregation to multiple layers to capture higher-order relationships recursively. At layer $l$, the updated representation is:


(7) \begin{equation*} {e}_h^{(l)}=f\left({e}_h^{\left(l-1\right)},{e}_{N_h}^{\left(l-1\right)}\right) \end{equation*}

(8) \begin{equation*} {e}_{N_h}^{\left(l-1\right)}=\sum_{\left(h,r, ta\right)\in{N}_h}\pi \left(h,r, ta\right){e}_{ta}^{\left(l-1\right)} \end{equation*}

where ${e}_{ta}^{\left(l-1\right)}$ represents $ta$ at the previous layer $\left(l-1\right)$, carrying information from its $\left(l-1\right)$-hop neighbors. At the initial layer, ${e}_h^0$ is set as ${e}_h$, which further contributes to the representation of element $h$ in layer $l$. By stacking multiple propagation layers, we ensure that biological context-based collaborative signals are effectively incorporated into the representation learning process.

After $L$ layers of propagation, we obtain multiple representations for the peptide node $p$, $\left\{{e}_p^{(1)},\dots, {e}_p^{(L)}\right\}$. Similar for the TCR node $t$, we obtain $\left\{{e}_t^{(1)},\dots, {e}_t^{(L)}\right\}$. Since the output of the $l$-th layer is the feature fusion of the tree structure with depth $l$ rooted at $p$ or $t$, different layer outputs emphasize connection information of different orders. Therefore, we use the layer-aggregation mechanism [30] to concatenate the representations of each layer into a single vector:


(9) \begin{equation*} {e}_p^{\ast }={e}_p^{(0)}\parallel \cdots \parallel{e}_p^{(L)},{e}_t^{\ast }={e}_t^{(0)}\parallel \cdots \parallel{e}_t^{(L)} \end{equation*}

where $\parallel$ demotes the concatenation operation. We can not only enrich the initial embeddings by embedding propagation, but also control the propagation strength by adjusting $L$.

### Construction of sequence-aware interaction preference module

To effectively capture TCR–peptide binding, we design a sequence-aware interaction preference module that integrates representation learning with self-attention mechanism and CNN-based interaction pattern extraction. This module quantifies interaction preferences and ultimately outputs a binding probability score.

The amino acid sequences of peptides and TCRs are first embedded using learnable embeddings. Next, we apply multi-layer self-attention to extract meaningful sequence features. To model interactions between peptides and TCRs, we define an aggregation function $F$ using the dot product:


(10) \begin{equation*} {I}_{i,j}=F\left({E}_p^P,{E}_t^T\right) \end{equation*}

where ${E}_p^P$ and ${E}_t^T$ represent the embeddings of peptides $p$ and TCRs$t$, respectively. The output tensor $I\in{R}^{\varTheta_t,{\varTheta}_p,\mathrm{\phi}}$ is obtained, where ${\varTheta}_t$ and ${\varTheta}_p$ represent the length of peptide and TCR, respectively, and $\phi$ is the output dimension of $F$. Each element of this tensor considers the interaction of substructure of peptide and TCR. Since the output of $F$ is one-dimensional for each pair, $I$ is transformed into a two-dimensional interaction map. Inspired by MolTrans [31], and considering the complexity of interactions between TCR–peptide pairs, the model also considers interactions in neighboring regions. A CNN layer further processes the interaction map [32]. To compute the interaction probability, we first perform a flatten operation on the features output by the convolutional layer. Then, a linear layer is applied to obtain the preference vector ${P}_{Pref}$ for TCR–peptide pairs based on peptide and TCR sequences.

### Construction of the prediction module

We concatenate ${P}_{Pref}$ with the layer-normalized embeddings of peptides and TCRs, ${e}_p^{\ast }$ and ${e}_t^{\ast }$, respectively, to form a new feature vector:


(11) \begin{equation*} h=\mathrm{concat}\left({P}_{pref},{e}_p^{\ast },{e}_t^{\ast}\right) \end{equation*}

where $concat\left(\bullet \right)$ denotes the concatenation operation of vectors. We then feed the concatenated vector $h$ into the prediction module ${f}_{\mathrm{Prediction}}$ to compute the final interaction probability score:


(12) \begin{equation*} {P}_{s\mathrm{core}}={f}_{\mathrm{Prediction}}(h) \end{equation*}

After obtaining ${P}_{\mathrm{Pref}}$ for positive and negative pairs based on peptide and TCR sequences, we compute the peptide embedding and the match differences between positive and negative TCR embeddings after performing a linear transformation on ${e}_p^{\ast }$ and ${e}_t^{\ast }$. The formula is:


(13) \begin{equation*} g\left(p,r,t\right)={\left|\left|{W}_p{e}_p^{\ast }+{P}_{Pref}-{W}_t{e}_t^{\ast}\right|\right|}_2^2 \end{equation*}

where $g\left(p,r,t\right)$ means the score of the positive samples $g{\left(p,r,t\right)}_{pos}$ and the negatives $g{\left(p,r,t\right)}_{neg}$.

### Model optimization

The model jointly optimizes the weights of the transformer encoder and CNN using the binary cross-entropy loss function with the true binding label $Y$:


(14) \begin{equation*} {\mathcal{L}}_{Pref}=-\left[ Ylog\left({P}_{score}\right)+\left(1-Y\right)\mathit{\log}\left(1-{P}_{score}\right)\right] \end{equation*}

After integrating features derived from both the CKG and sequence data, we compute the logarithmic sigmoid of the score differences between positive and negative samples, and then use this to obtain the CKG loss. The loss function is as follows:


(15) \begin{equation*} {\mathcal{L}}_{CF}=\sum_{\left(p,{t}_{,}j\right)\in O}-\mathit{\ln}\sigma \left(g{\left(p,r,t\right)}_{pos}-g{\left(p,r,t\right)}_{neg}\right) \end{equation*}

where $O=\left\{\left(p,t,j\right)|\left(p,t\right)\in{R}^{+},\Big(p,j\Big)\in{R}^{-}\right\}$ denotes the training set, ${R}^{+}$ denotes the observed (positive) interaction between peptide $p$ and TCR $t$, and ${R}^{-}$ means the unobserved (negative) interaction between peptide $p$ and TCR $j$. $\sigma \left(\cdotp \right)$ is the sigmoid function.

Finally, objective function jointly learning Equation (2), Equation (14), and Equation (15) is:


(16) \begin{equation*} {\mathcal{L}}_{CKG- TPI}={\mathcal{L}}_{KG}+{\mathcal{L}}_{Pref}+{\mathcal{L}}_{CF} \end{equation*}

The training process and parameter settings are described in the Supplementary Materials and Supplementary Table S1.

### Data collection and preprocessing

**Figure 2:**
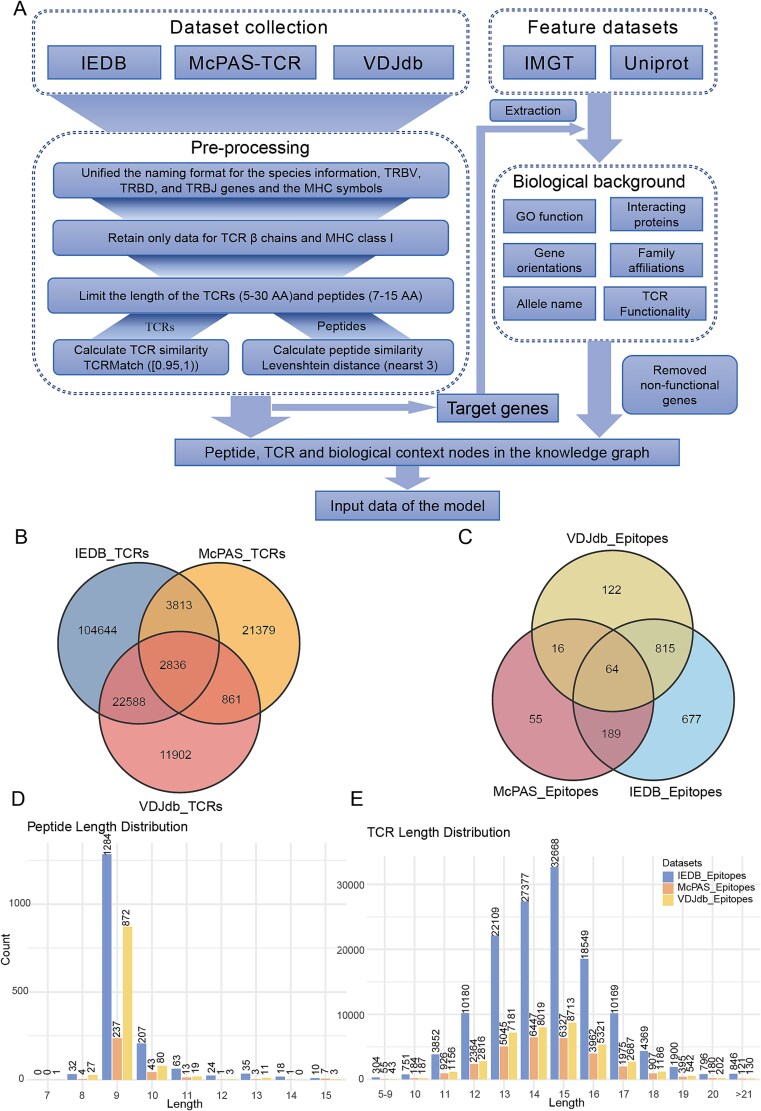
Overview of data processing workflow and dataset statistics. (A) Schematic of the data processing workflow. (B) and (C) Distribution of TCRs and peptides across the IEDB, VDJdb, and McPAS-TCR datasets. (D) and (E) Length distribution of peptides and TCRs in the preprocessed datasets, respectively.

In this study, data were collected from three publicly available databases containing experimentally validated TCR–peptide binding sequences, IEDB [33], VDJdb [34], and McPAS-TCR (McPAS) [35]. To ensure high data quality and applicability, we followed the data filter settings used in several state-of-the-art TCR–peptide prediction studies [11, 36, 37]. After rigorous screening, the IEDB dataset retained 181 381 unique CDR3-peptide pairs, involving 133 881 TCRs assigned to 1745 peptides. The VDJdb dataset retained 57 705 unique CDR3-peptide pairs, involving 38 187 TCRs mapped to 1017 peptides. The McPAS-TCR dataset retained 30 937 unique CDR3-peptide pairs, containing 28 889 TCRs linked to 324 peptides. A comprehensive overview of the dataset and the preprocessing procedure is presented in Fig. 2A and further detailed in the Supplementary Materials under the sections “Detailed data description” and “Data preprocessing.” After filtering, the distributions of TCRs and peptide counts in the three datasets are presented in Fig. 2B and C, while their sequence length distributions are shown in Fig. 2D and E.

### Generation of negative datasets

In TCR–peptide studies, five-fold cross-validation is commonly used to evaluate model performance [11, 36]. Since all TCR–peptide pairs in the original datasets are known matched samples (positive samples), it is necessary to generate negative samples to train a generalized model that helps the model learn the key features essential for accurate prediction. Specifically, negative samples are generated by mismatching the antigen peptide with the TCR sequences [11, 38]. This study employs two negative sampling strategies: random TCR sampling (Random TCR) and strict TCR sampling (Strict TCR). Random TCR generates negative data by randomly selecting TCRs from the pool of known positive pairs and pairing them with peptides they are not known to bind. All known positive pairs are excluded during sampling. Strict TCR divides the dataset into five non-overlapping folds based on unique TCRs, ensuring that each fold contains distinct TCRs. For each fold, TCRs in negative samples are randomly generated from TCRs in the positive samples to avoid overlap of negative samples between the training and test sets. This strategy is more stringent and better assesses the model’s generalization ability to unseen TCR sequences. We generated negative samples at 10 times the number of positive samples for each dataset.

## Results

### Comparisons with state-of-the-art methods and cross-dataset validation

**Figure 3:**
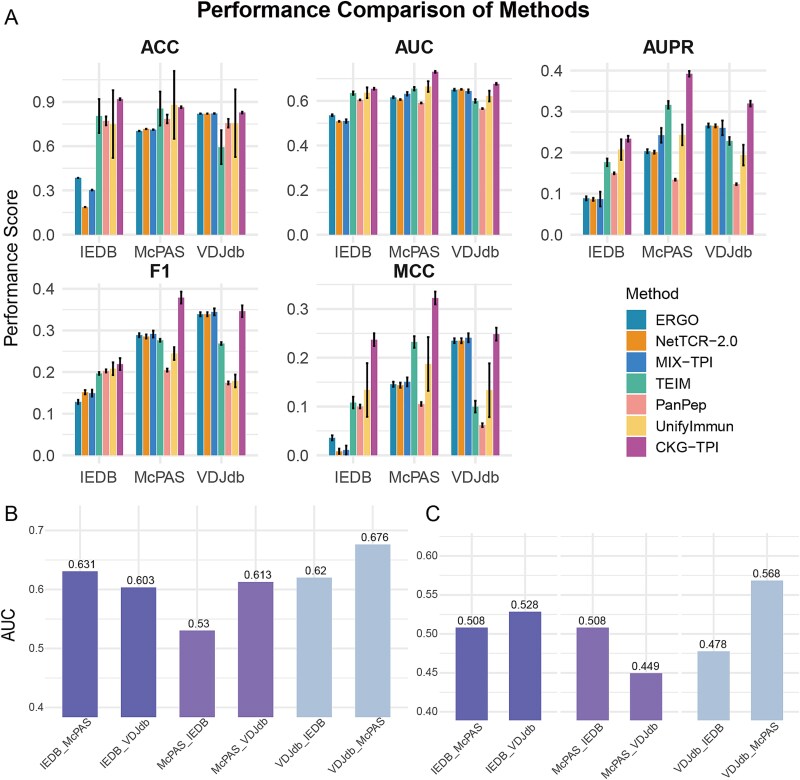
Comparison of model performance. (A) Comparison of performance indicators for seven methods on the IEDB, McPAS, and VDJdb datasets. Each legend in the figure represents a different method. (B) and (C) AUC performance of CKG-TPI and sequence-based strong baseline model TEIM under cross-dataset validation. In each figure, the training sets are indicated by the dataset name shown before the underscore on the x-axis labels.

To evaluate the performance of CKG-TPI, we conducted five-fold cross-validation on three CDR3β datasets: IEDB, McPAS, and VDJdb, and compared the results with six state-of-the-art baseline models: ERGO, NetTCR-2.0, TEIM, and MIX-TPI, PanPep, and UnifyImmun. For fairness, all baseline models were configured according to their default parameters as reported in the respective original publications, and were restricted to TCR β-chain and peptide sequence data as input. The comparative performance of CKG-TPI and the baseline models across all three datasets is summarized in Fig. 3A. Focusing on McPAS dataset where overall model performance was relatively stronger, CKG-TPI achieved an average AUC of 0.7303, outperforming the best-performing baseline, UnifyImmun (AUC = 0.6646) by 9.89%. In terms of AUPR, CKG-TPI reached 0.3921, reflecting a 23.93% improvement over TEIM (AUPR = 0.3164), the strongest baseline on this metric. Beyond accuracy, CKG-TPI also demonstrated superior performance in both F1 score (0.3788) and MCC (0.3224). Compared to MIX-TPI and TEIM, the strongest baselines for F1 and MCC respectively, CKG-TPI achieved a 30.04% gain in F1 score (MIX-TPI: 0.2913) and a 27.92% improvement in MCC (TEIM: 0.2324). The complete performance metrics are summarized in Table S2, and the statistical significance of performance differences between methods is illustrated in Supplementary Figure S5. These results clearly demonstrate that CKG-TPI achieves substantial performance gains over baseline models. The improvement is primarily attributed to the incorporation of biologically relevant auxiliary entities via the CKG. By dynamically updating node representations through information propagation and aggregation, the model can more accurately capture the specificity of TCR–peptide interactions.

To further assess the model’s robustness and generalization ability, we conducted cross-dataset validation across the VDJdb, McPAS, and IEDB datasets. For each configuration, a model was trained on one dataset and evaluated on the remaining two, using Strict TCR to ensure that TCRs in the test set were absent from the training set. This resulted in six train-test configurations: VDJdb-McPAS, VDJdb-IEDB, McPAS-VDJdb, McPAS-IEDB, IEDB-VDJdb, and IEDB-McPAS, where the first dataset was used for training and the second for testing. In VDJdb-McPAS setting, CKG-TPI achieved the highest AUC of 0.6760 (Fig. 3B), nearly identical to the within-dataset cross-validation performance on VDJdb (AUC = 0.6768), suggesting that our model exhibits robustness despite data distribution shifts. Across all configurations, CKG-TPI maintained an average AUC above 0.6121, significantly outperforming the strong sequence-based model TEIM (AUC ~ 0.5 (mean AUC = 0.5067), equivalent to random guessing, see Fig. 3C). These results demonstrate the effectiveness of incorporating collaborative biological context through CKG for enhancing the generalizability of TCR–peptide binding prediction.

### Contribution of the CKG and attention weight visualization

Unlike previous methods that solely relied on TCR and peptide sequences, or even those incorporated amino acid physicochemical features, CKG-TPI introduces a novel CKG to provide biological context associated with TCRs and peptides. To evaluate the contribution of CKG, we designed two model variants: CKG-only (CKG-TPI_only_graph) and Sequence-only (CKG-TPI_only_seq). The former utilizes only knowledge graph without sequence information, while the latter uses only TCR and peptide sequences.

**Figure 4:**
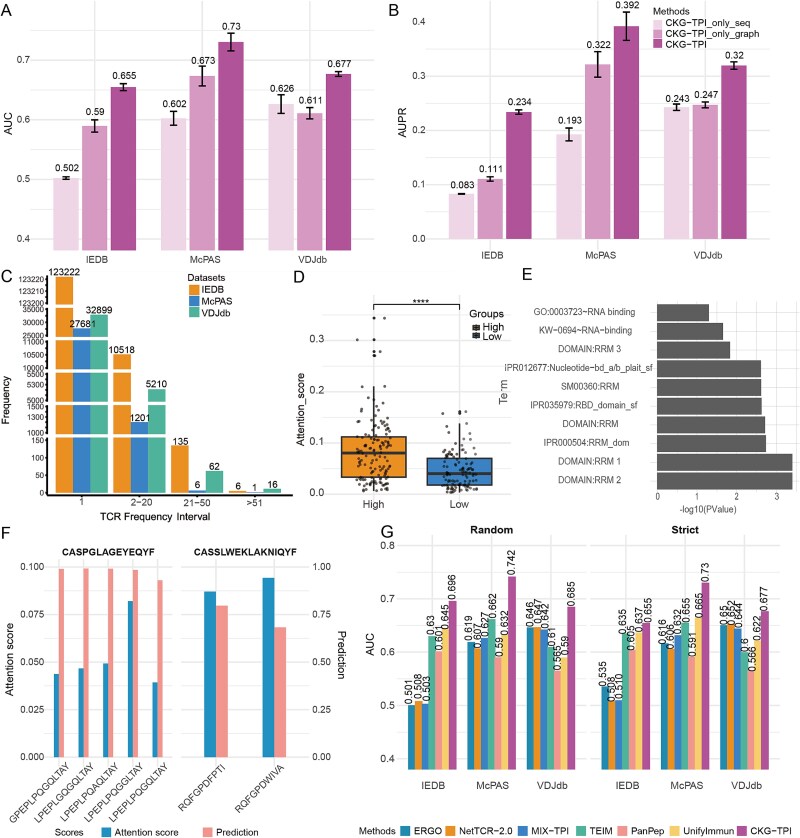
Visualization of CKG contribution, attention scores and method comparison under different negative sampling strategies. (A) and (B) Comparative AUC and AUPR performance of CKG-TPI and its two variants. (C) Frequency distribution of TCRs across the three datasets, with frequencies binned into preset intervals. (D) Attention scores for high and low TCR attention groups. The groups are labeled “High” and “Low” in the figure. “^****^” indicates *P*-value <.0001. (E) Functional enrichment analysis of peptides with high attention scores. (f) Distribution of attention weights and predicted scores for cross-reactivity TCRs and binding peptides. (G) AUC comparison between CKG-TPI and the six baseline methods under random TCR and strict TCR strategies.

Evaluation results across all three independent datasets showed that CKG-TPI outperformed both variants (Fig. 4A and B, Supplementary Figure S6). In the McPAS dataset, the AUC (0.7303) and AUPR (0.3921) of CKG-TPI were 8.47% and 21.88% higher than CKG-only (AUC: 0.6733, AUPR: 0.3217), respectively. Compared to Sequence-only (AUC: 0.6025, AUPR: 0.1926), the improvements were even more substantial: 21.21% in AUC and 103.58% in AUPR. Furthermore, the CKG-only model outperformed all sequence-based baselines, highlighting the value of the biological context provided by the CKG. In the IEDB dataset, CKG-only also outperformed several sequence-based baseline methods, including ERGO, NetTCR-2.0, and MIX-TPI (Supplementary Figs S7 and S8). Specifically, it achieved an AUC 6.59% higher and an AUPR 32.82% higher than the best-performing baseline MIX-TPI. These findings underscore that the integration of sequence-level and CKG-based information enables CKG-TPI to effectively capture the complex TCR–peptide binding patterns.

To further interpret the model’s decision-making, we visualized the attention weights learned in the CKG. The graph attention mechanism dynamically assigns weights among nodes, facilitating efficient information propagation across nodes with higher-order connectivity. Leveraging these learned attention weights, we inferred the TCR–peptide binding bias. As a case study, we analyzed attention patterns for cross-reactive TCRs, a well-known phenomenon in the immune response, which are capable of binding to multiple distinct peptides. Cross-reactivity TCRs were frequently observed across all three datasets (Fig. 4C), with the majority occurring in the frequency range of 2–20 distinct binding peptides. Based on the model trained on VDJdb, we calculated attention scores for TCR–peptide pairs in the test set. TCRs were stratified into high-attention and low-attention groups based on the median attention score. A Wilcoxon rank-sum test confirmed a statistically significant difference between the two groups (*P* = 1.827 × 10^−8^, Fig. 4D).

To realize the biological interpretability, we performed gene annotation for peptides with high attention scores and found many of the corresponding genes were immune-related, including IGF2BP2 [39], TYR [40], ELAVL4 [41, 42], and U2AF2 [43]. Functional enrichment analysis of these genes revealed that the enriched terms include multiple RNA recognition motifs (RRMs 1, 2, 3), RNA-binding domains, and Gene Ontology terms associated with RNA binding, all of which are closely linked to post-transcriptional regulation and immune processes (Fig. 4E) [44–46]. These results demonstrate that attention weights learned by the model are biologically meaningful.

Further analysis of TCR–peptide binding pairs showed that attention scores vary across peptides recognized by the same cross-reactive TCR (Fig. 4F). Within the knowledge graph, certain peptides recognized by a cross-reactivity TCR received higher attention scores, which aids in identifying peptides with stronger specificity for the TCRs. These peptide-TCR pairs may serve as prioritized candidates for experimental validation, potentially accelerating the development of targeted immunotherapies.

### Comparison of the methods under different negative sampling strategies

To assess the robustness of CKG-TPI under varying training conditions, we evaluated model performance using both Random TCR and Strict TCR sampling strategies across the IEDB, McPAS, and VDJdb datasets. Results showed that for most sequence-based models, AUC scores experienced slight performance improvements under stricter sampling, models incorporating additional features, including CKG-TPI, generally exhibited a modest decline in AUC, particularly on the IEDB dataset, where the drop reached 6.31% (Supplementary Figure S9). Despite this decline, CKG-TPI consistently outperformed all six baseline models under both sampling strategies (Fig. 4G; Supplementary Figure S10). Given the risk of data leakage in Random TCR, where identical TCRs may appear in both training and test sets, potentially inflating performance metrics. Considering the scientific rigor required for TCR analysis, we recommend Strict TCR in experimental design.

### Screening of peptide-specific immune-responsive T cells

Autoimmune diseases arise from immune system dysfunction, where T cells aberrantly recognize and attack self-antigen peptides, leading to pathological damage [47]. In tumor immunotherapy, adoptive cell transfer therapy (ACT), has emerged as a promising strategy, relying on the precise selection and expansion of T cells that specifically recognize tumor neoantigens [48]. Effective identification of T cells targeting antigens is crucial for both autoimmune disease control and the development of novel cancer immunotherapies.

In this study, 125 experimentally validated peptide–TCR pairs (including tumor neoantigen peptide–TCR pairs) were integrated [49–52]. After removing duplicate entries overlapping with the VDJdb, McPAS, and IEDB datasets, the remaining 72 peptide-TCR pairs were used to evaluate the model’s capability to identify immune-responsive T cells. We combined the experimentally validated TCRs with all TCRs from the original dataset and removed duplicates. The binding probability of each TCR with each peptide was then calculated and ranked across all models. Higher-ranked TCRs indicate stronger peptide binding probability.

**Figure 5:**
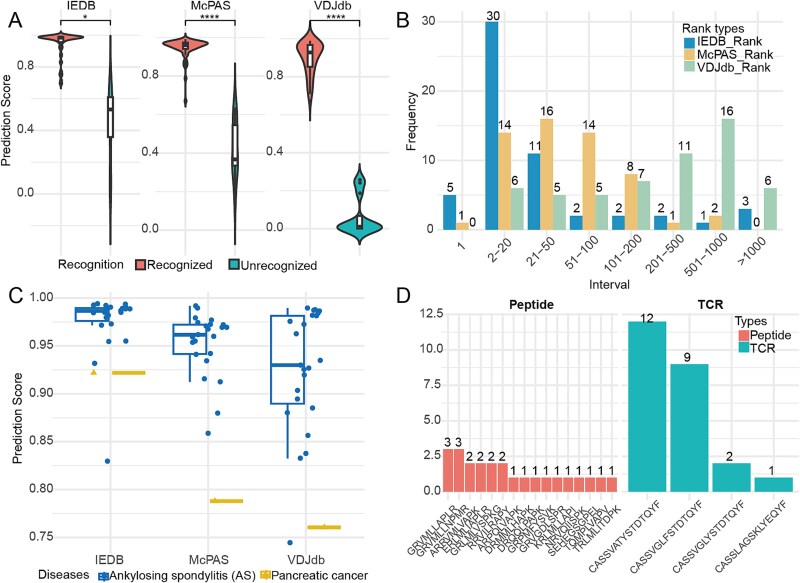
Overview of scores and rankings for peptide-specific immune responsive T cells. (A) Identification of the critical threshold distinguishing recognized and unrecognized groups, based on t-test significance across different data-trained models. “^**^” denotes *P*-value <.01, and “^****^” denotes *P*-value <.0001. (B) Rank distribution of 56 peptide-TCR pairs within the recognized group under three prediction frameworks trained on IEDB, McPAS, and VDJdb datasets, respectively. (C) Predicted values of unseen antigenic peptides across various diseases under different prediction models. Circles represent ankylosing spondylitis, while triangles indicate pancreatic cancer. (D) Illustration of immune response cross-reactivity, highlighting the potential for shared recognition between different antigenic peptides.

The results showed that 77.78% of the experimentally verified TCR–peptide pairs were accurately identified (prediction score > 0.65, Fig. 5A). Statistical analysis of the rank distribution revealed that 10.71% (6 out of 56) of the identified TCR–peptide pairs were ranked first (rank = 1; Fig. 5B), indicating that the model exhibits relatively predictive accuracy and holds potential for providing promising candidate TCRs for cancer immunotherapy development. Moreover, CKG-TPI successfully identified TCRs with binding specificity to 24 previously unseen antigenic peptides among three datasets, further highlighting its generalization capability and potential for immune-responsive T cell screening (Fig. 5C and D).

## Discussion

In recent years, TCR-based immunotherapy strategies have achieved promising clinical outcomes in the treatment of unresectable or metastatic uveal melanoma and rare soft tissue cancers [53, 54]. Therefore, accurate prediction of TCR–peptide binding not only deepens our understanding of the molecular mechanisms underlying immune recognition but also has the potential to accelerate advancements in immunotherapy, providing new tools and strategies for personalized medicine.

With the continuous accumulation of publicly available TCR–peptide binding data, data-driven machine learning-based predictive models [55, 56] have emerged as a research hotspot. However, previous studies have primarily relied on sequences or physicochemical features, without fully incorporating knowledge network information related to TCRs and peptides. To address this limitation, we propose CKG-TPI, a novel predictive framework based on a CKG-based attention network. Through layer-by-layer propagation and aggregation, CKG-TPI dynamically updates representations of TCRs and peptides while integrating a sequence-aware interaction preference module to produce refined predictions of TCR–peptide binding specificity.

The primary advantage of CKG-TPI lies in its ability to simultaneously capture local relationships between TCR, peptide sequences, and global semantic information from knowledge graphs, enabling precise prediction of TCR–peptide binding. Compared to the best-performing baseline model, CKG-TPI achieved improvements of 9.89% in AUC and 23.93% in AUPR. It also showed superior performance in both F1 score (0.3788) and MCC (0.3224), significantly outperforming the second-best method MIX-TPI 30.04% (F1 score: 0.2913) and TEIM 27.92% (MCC: 0.2324), respectively. Moreover, the model demonstrated strong robustness and generalization ability in cross-dataset validation, with the highest AUC of 0.6760 (VDJdb-McPAS), which was nearly identical to its five-fold cross validation result (0.6768). In terms of unseen antigenic peptides recognition, CKG-TPI accurately identified six peptide-TCR binding pairs from a large TCR repertoire. Moreover, its attention weight visualization provides valuable insights into the binding biases of cross-reactivity TCRs, further enhancing our understanding of TCR–peptide binding. These findings suggest that CKG-TPI has significant potential as a new tool for personalized medicine.

Despite its strong performance, CKG-TPI has certain limitations. First, due to the scarcity of TCR α-chain data, the current model does not integrate α-chain information into the CKG or include it as matching information for TCR β-chains in the interaction prediction module. This omission may introduce biases when predicting certain TCR–peptide binding. Second, although the knowledge graph can capture high-order biological features, its construction and optimization heavily rely on high-quality data annotation and domain expertise, which could affect the model’s generalizability in scenarios with sparse or noisy data.

To address these limitations, future research may explore more efficient methods for knowledge graph construction, incorporating richer biological context to enhance the generalization capability of the model. As TCR α-chain sequencing data becomes increasingly available, it would be promising to integrate the α-chain into the model, either through expanded knowledge graphs or multi-chain representation learning, enabling more comprehensive modeling of TCR specificity, thereby improving predictive accuracy, especially for cross-individual generalization. Furthermore, the proposed CKG-TPI framework can be extended to broader immunological applications, such as neoantigen screening, autoimmune epitope identification, and personalized cancer vaccine design, further enhancing its potential impact in clinical medicine.

**Key Points**

- CKG-TPI first proposes a collaborative knowledge graph-based attention network within the framework of graph neural networks for accurate prediction of the binding specificity between TCRs and peptides.
- CKG-TPI is the first method that constructs a comprehensive knowledge graph by incorporating amino acid sequences and biological context of both peptides and TCRs to predict TCR–peptide binding specificity.
- CKG-TPI harnesses hierarchical neighbor propagation and dynamic importance aggregation to capture the complexity of high-order relationships between nodes, and further incorporates a preference scoring mechanism for TCR–peptide binding, which significantly enhances the model’s performance.
- CKG-TPI exhibits validity, robustness, and interpretability in the importance of higher-order relationships in the knowledge graph.

## Supplementary Material

Supplementary_materials_bbaf486

## Data Availability

CKG-TPI is implemented in Python using Pytorch. All the data and code are available at https://github.com/Boyahankuke/CKG-TPI.
