# Supplementary material for: CKG-TPI: integrating collaborative knowledge graph with sequence interactions for TCR–peptide binding specificity
Source: Brief Bioinform. 2025 Sep 22;26(5):bbaf486. doi: 10.1093/bib/bbaf486 (PMC12451105; doi:10.1093/bib/bbaf486)
Supplement: Supplementary_materials_bbaf486 [file supplementary_materials_bbaf486.docx]

**CKG-TPI: integrating collaborative knowledge graph with sequence interactions for TCR-peptide binding specificity**

**Supplementary Materials**

Yue Liu^1,#^, Haoyan Wang^1,#^, Guohua Wang^1^, Tao Jiang^1,2,*^, Yadong Wang^1,2,*^ and Yadong Liu^1,2,*^

^1^ Faculty of Computing, Harbin Institute of Technology, Harbin, Heilongjiang, 150001, China

^2^ Zhengzhou Research Institute, Harbin Institute of Technology, Zhengzhou, Henan, 450000, China

^*^Corresponding authors: Yadong Wang: [ydwang@hit.edu.cn](mailto:ydwang@hit.edu.cn)

^#^Joint first authors: Yue Liu and Haoyan Wang contributed equally to this work.

Content

[Supplementary Notes 3](#_Toc202650408)

[Detailed data description 3](#_Toc202650409)

[Data preprocessing 3](#_Toc202650410)

[Parameter setting 4](#_Toc202650411)

[Performance evaluation 5](#_Toc202650412)

[Figure S1. The distribution of similarity quantity by interval statistics. 6](#_Toc202650413)

[Figure S2. Performance evaluation results under five different peptide Levenshtein distance thresholds (1, 2, 3, 4, and 5) across three independent datasets. 7](#_Toc202650414)

[Figure S3. Performance comparison between models with unpruned nodes and those retaining only the top three ranked nodes (top-3-pruned). 8](#_Toc202650415)

[Figure S4. Illustration of auxiliary features used in the construction of the CKG. 9](#_Toc202650416)

[Figure S5. Statistical significance (t-test) results of performance metrics (AUC, AUPR, ACC, F1-score, and MCC) among all methods across three independent datasets. 10](#_Toc202650417)

[Figure S6. Comparison of AUC and AUPR among CKG-TPI and its two variants. 11](#_Toc202650418)

[Figure S7. AUC comparison between CKG-TPI_only_graph and baseline methods. 12](#_Toc202650419)

[Figure S8. Comparison of AUPR between CKG-TPI_only_graph and baseline methods. 13](#_Toc202650420)

[Figure S9. Performance of CKG-TPI under two different TCR sampling strategies. 14](#_Toc202650421)

[Figure S10. AUPR performance of CKG-TPI compared to four baseline methods under two TCR sampling strategies. 15](#_Toc202650422)

[Table S1. Parameter settings. 16](#_Toc202650423)

[Table S2. Performance comparison between CKG-TPI and other state-of-the-art models. 17](#_Toc202650424)

[Reference 18](#_Toc202650425)

**Supplementary Notes**

**Detailed data description**

The IEDB is a free resource containing a comprehensive collection of 206,418 experimental data on antibodies and T-cell epitopes associated with infectious diseases, allergies, autoimmunity, and transplantation in humans, non-human primates, and other animal species. VDJdb is a collated TCR sequence database with known antigen specificities containing 89,106 pairs of CDR3 sequences, including the α and β chains of TCR, involving three species: human, monkey and mouse. McPAS-TCR is a manually curated catalogue of TCR sequences linking TCR sequences to their antigen targets or related pathologies and organs, containing 39,985 pairs of sequences from mouse and humans. These databases contain information on experimentally validated T-cell receptor (TCR) and peptide binding.

**Data preprocessing**

To ensure the high quality and applicability of the three publicly available datasets, we followed the experimental setting of the state-of-the-art TCR-peptide prediction methods and processed the data as follows [1-3]. Given that approximately 95% of T cells in humans are αβ T-lymphocytes expressing TCR α and β chains [4, 5]. During T cell maturation, the variable (V) gene, diversity (D) gene, and joining (J) gene segments of the β chain are randomly recombined to form a functional V segment [6], whereas for the α chain, only the V and J gene segments are recombined, without the D gene [7, 8]. Studies have shown that the CDR3 β sequence may play a more important role in determining the specificity of TCR binding to epitopes [9, 10]. Therefore, for the three datasets, we first unified the naming format for the species information, TRBV, TRBD, and TRBJ genes as well as the MHC symbols. We retained only the entries corresponding to the β chain of the TCRs, and MHC-I class entries for humans. We then limited the length of CDR3 to between 5 and 30 amino acids, and the length of antigen epitopes to between 7 and 15 amino acids according to the length distribution patterns of CDR3 and peptides. For each remaining TCR, we retained the following information: the amino acid sequence of the CDR3 β chain, the epitope amino acid sequence, and information on βV, βJ, and MHC genes of any precision. We unified the symbols for all V and J genes. After screening, the IEDB dataset retained 181,381 unique CDR3-epitope pairs, involving 133,881 TCRs assigned to 1,745 epitopes. The VDJdb dataset retained 57,705 unique CDR3-epitope pairs, involving 38,187 TCRs mapped to 1,017 epitopes. The McPAS-TCR database retained 30,937 unique CDR3-epitope pairs, containing 28,889 TCRs assigned to 324 epitopes.

To enhance the connectivity between TCRs and reduce sparsity in the constructed knowledge graph, we use the TCRMatch tool to calculate the similarity between TCRs [11]. A threshold of [0.95, 1) is applied to filter TCRs, retaining those with high similarity. Considering the threshold settings in the TCRMatch study and the distribution characteristics of similarity scores in our work, we applied a threshold of [0.95, 1] to filter and retain highly similar TCRs. The interval distribution of TCR similarity scores is shown in Supplementary Figure S1. Similarity between peptides were calculated with Levenshtein distance method with a threshold of 3 and select the three peptides closest to the current peptide as the retained peptide nodes [12]. The threshold was selected based on experimental evaluations conducted on three independent datasets, where five different edit distance thresholds (1, 2, 3, 4, and 5) were tested. As shown in Supplementary Figure S2, the results indicate that a threshold of 3 yields relatively better performance across datasets. To reduce the complexity of the constructed graph without significantly sacrificing model performance, we retained only the top three most similar peptide nodes for each node during graph construction. A paired t-test was conducted to compare model performance between the unpruned graph and the pruned version (top-3 similar nodes retained), showing no statistically significant difference in AUC (p = 0.877), AUPR (p = 0.470), ACC (p = 0.340), F1 (p = 0.709), and MCC (p = 0.555). As shown in Supplementary Figure S3, the performance of the model with top-3 pruning was slightly better overall, suggesting that this strategy not only improves computational efficiency but may also contribute modestly to performance enhancement.

In order to comprehensively obtain key information regarding TCR-peptide interactions, in addition to species, MHC and TCR coding information, we further searched for feature attributes related to TCR and peptide to enrich their respective knowledge graph feature structures. According to the TCR gene feature table from IMGT [13, 14], the encoding genes of TCRs can be functionally categorized into functional genes, non-functional genes, pseudogenes, and open reading frames (ORFs). We removed data entries with non-functional genes according to the IMGT annotations [15]. Different TCR genes also have different gene orientations. The GO functions of the target proteins to which the epitopes belong, along with information on interacting proteins and family affiliations, are also critical feature information. To facilitate the identification of available feature information for target proteins, we used the common target genes across various data resources as keywords. First, for each data resource, we added a column containing target gene information based on the name of the target protein. Then, by leveraging the gene names from the UniProt database [16, 17] and the official symbols and aliases listed in NCBI Gene (https://www.ncbi.nlm.nih.gov/gene/), we standardized the encoding genes for the target proteins. The epitope annotation information obtained from UniProt and the TCR information annotated from IMGT were used to construct the knowledge graph, seen in Supplementary Figure S4. Functional enrichment analysis was performed using the DAVID tool [18, 19], with a significance threshold set at p < 0.05.

**Parameter setting**

To ensure a fair and valid comparison, all models, including our proposed method, baseline algorithms as well as ablation experiments, were trained and evaluated on the same dataset splits. The data partitioning was performed once using a fixed random seed, and all models were trained using this identical split. During training, we alternately optimize the collaborative filtering loss $L_{CF}$ and the knowledge graph loss $L_{KG}$, and adopt the mini-batch Adam optimization algorithm [20] to optimize the embedding loss and prediction loss. Adam is a widely used optimizer capable of adaptively controlling the learning rate according to the absolute value of the gradient. Specifically, for a batch of randomly sampled $(h,r,ta,{ta}^{'})$, we update the embeddings for all nodes. Then, we randomly sample a batch of $(p,t,j)$, retrieve their representation after $L$ layers of propagation, and use the gradients of the prediction loss to update the model parameters (Table S1). All experiments were conducted using the following hardware and software configuration: GPU: NVIDIA RTX 4090 with 24 GB VRAM, CUDA version: 11.8.0, Python version: 3.8, PyTorch version: 2.0.0, CPU: AMD EPYC 7702 64-Core Processor (80 cores), RAM: 300 GB. To evaluate the computational cost of our method, we recorded the training time on three benchmark datasets. The average time per epoch and the total training time (over 50 epochs) are summarized as follows: McPAS dataset: ~180 seconds/epoch, total ~150 minutes, VDJdb dataset: ~300 seconds/epoch, total ~250 minutes, IEDB dataset: ~800 seconds/epoch, total ~666 minutes. These times include the full training process, including forward propagation, backpropagation, and validation evaluation. Overall, our method demonstrates acceptable computational efficiency and can be feasibly trained on widely available high-performance computing platforms.

**Performance evaluation**

The methods compared in this study were evaluated using two key metrics: the area under the receiver operating characteristic curve (AUC) and the area under the precision-recall curve (AUPR), with the results averaged over five-fold cross-validation. The ROC curve's x-axis represents the False Positive Rate (FPR), while the y-axis represents the True Positive Rate (TPR). The calculation formula is as follows:

$$FPR=\frac{FP}{TN+FP}$$

$$TPR=\frac{TP}{TP+FN}$$

AUPR measures the overall performance of precision and recall. The PR curve's x-axis represents Recall (also known as sensitivity), while the y-axis represents Precision. The specific formula is as follows:

$$PRE=\frac{TP}{TP+FP}$$

$$SEN=\frac{TP}{TP+FN}$$

where $TP$, $FP$, $TN$ and $FN$ represent the numbers of true positive, false positive, true negative and false negative, respectively. AUC reflects the model’s ability to distinguish between positive and negative samples. AUPR is suitable for imbalanced datasets, and enables to assess the model’s performance on the minority class. Compared to the ROC curve, the AUPR curve is more sensitive to the minority class (positive class). In addition, we employed multiple evaluation metrics, including Accuracy (ACC), F1-score, and Matthews Correlation Coefficient (MCC), to comprehensively assess the model performance in terms of prediction accuracy, positive class identification ability, and overall classification balance.

**
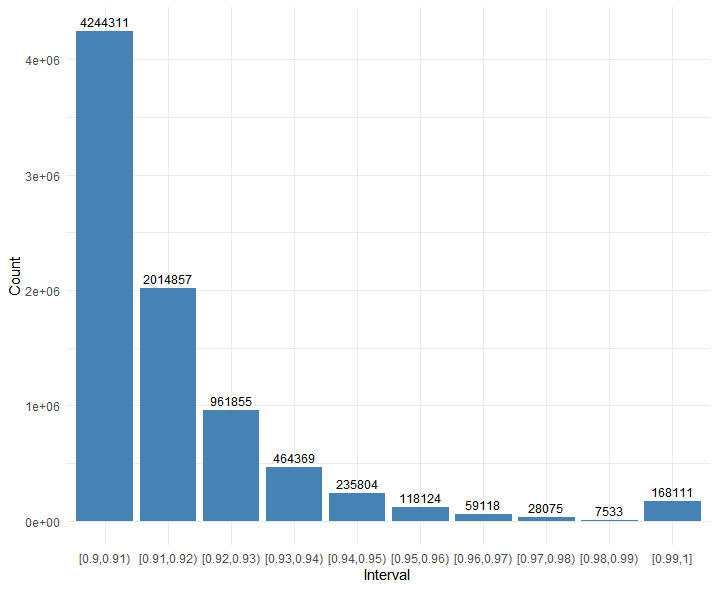
**

**Figure S1. The distribution of similarity quantity by interval statistics.**

**
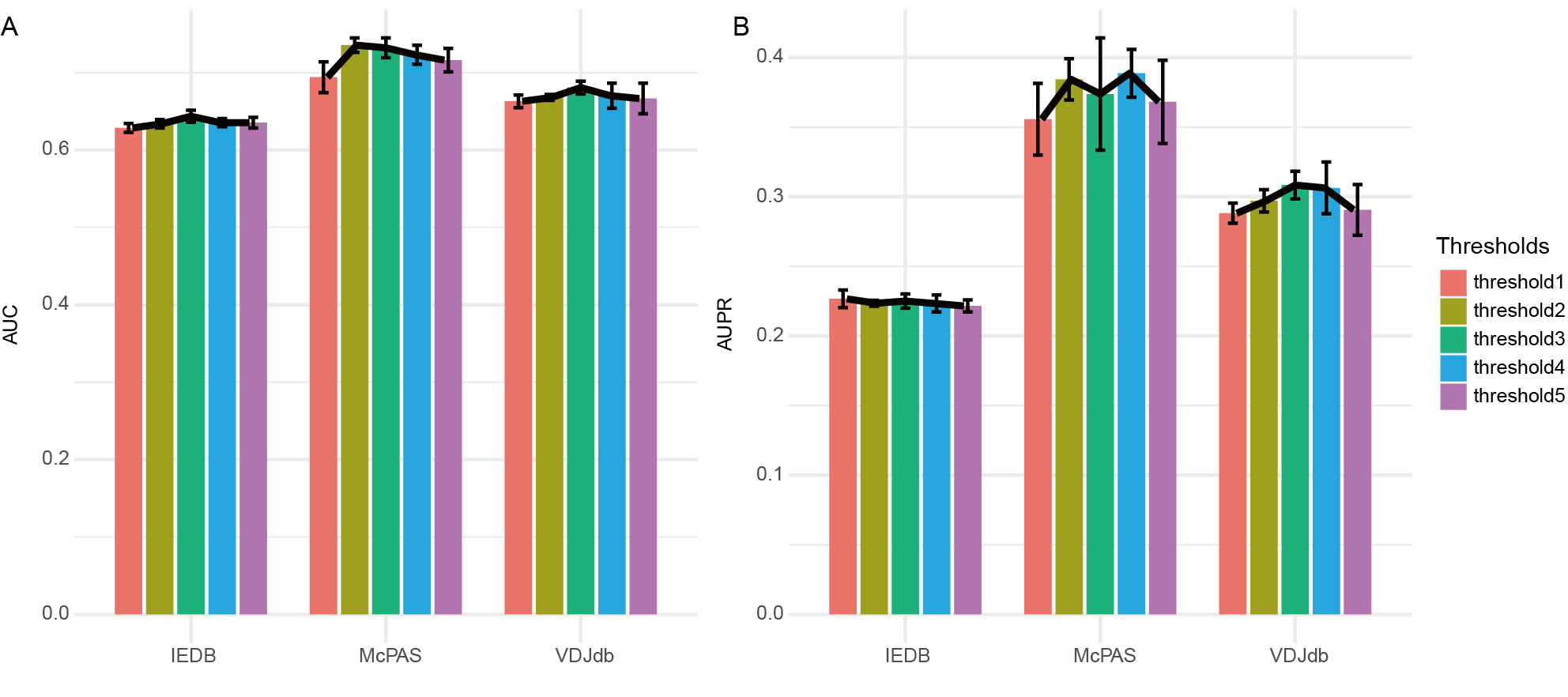
**

**Figure S2. Performance evaluation results under five different peptide Levenshtein distance thresholds (1, 2, 3, 4, and 5) across three independent datasets.**

**
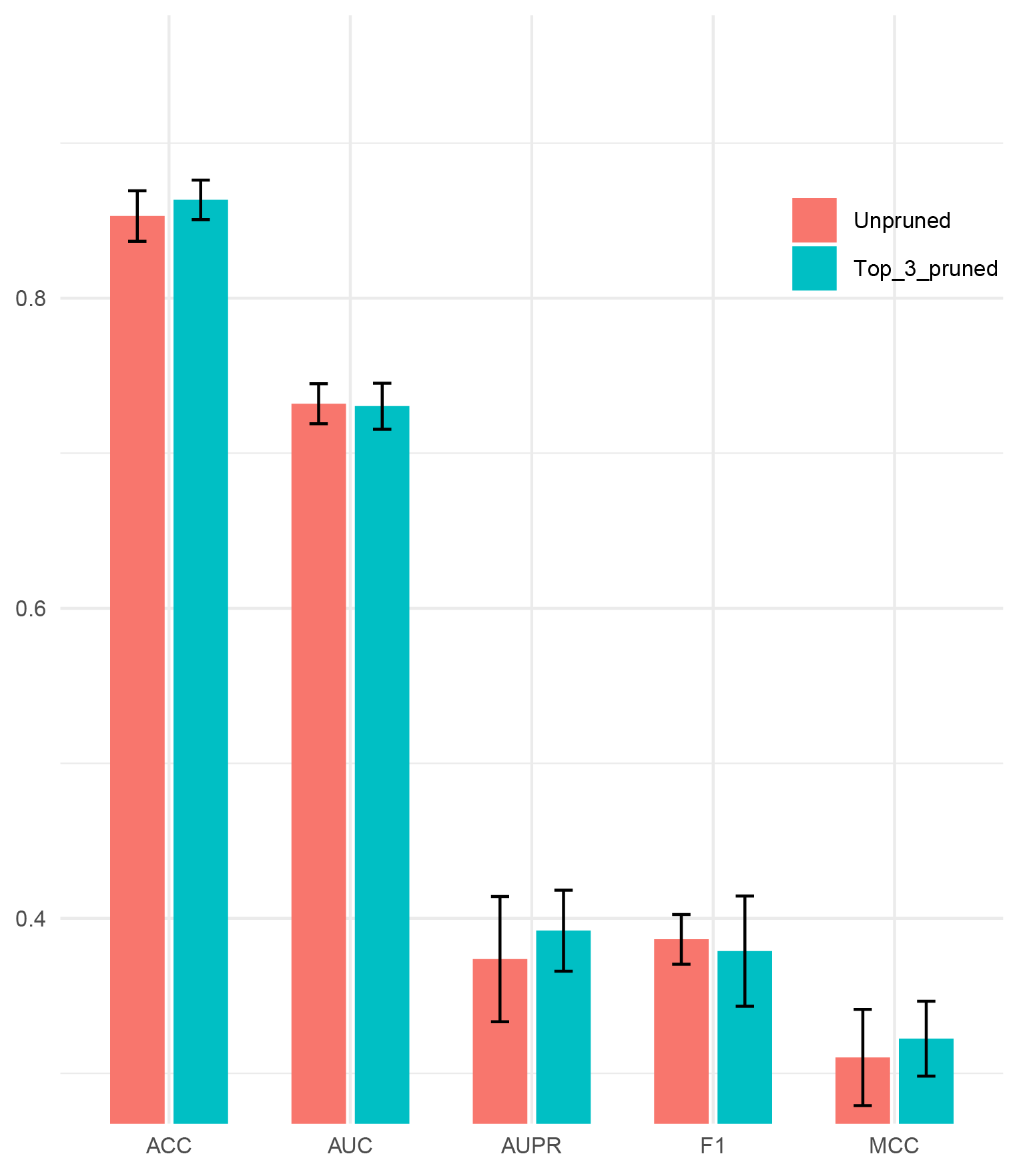
**

**Figure S3. Performance comparison between models with unpruned nodes and those retaining only the top three ranked nodes (top-3-pruned).**

**
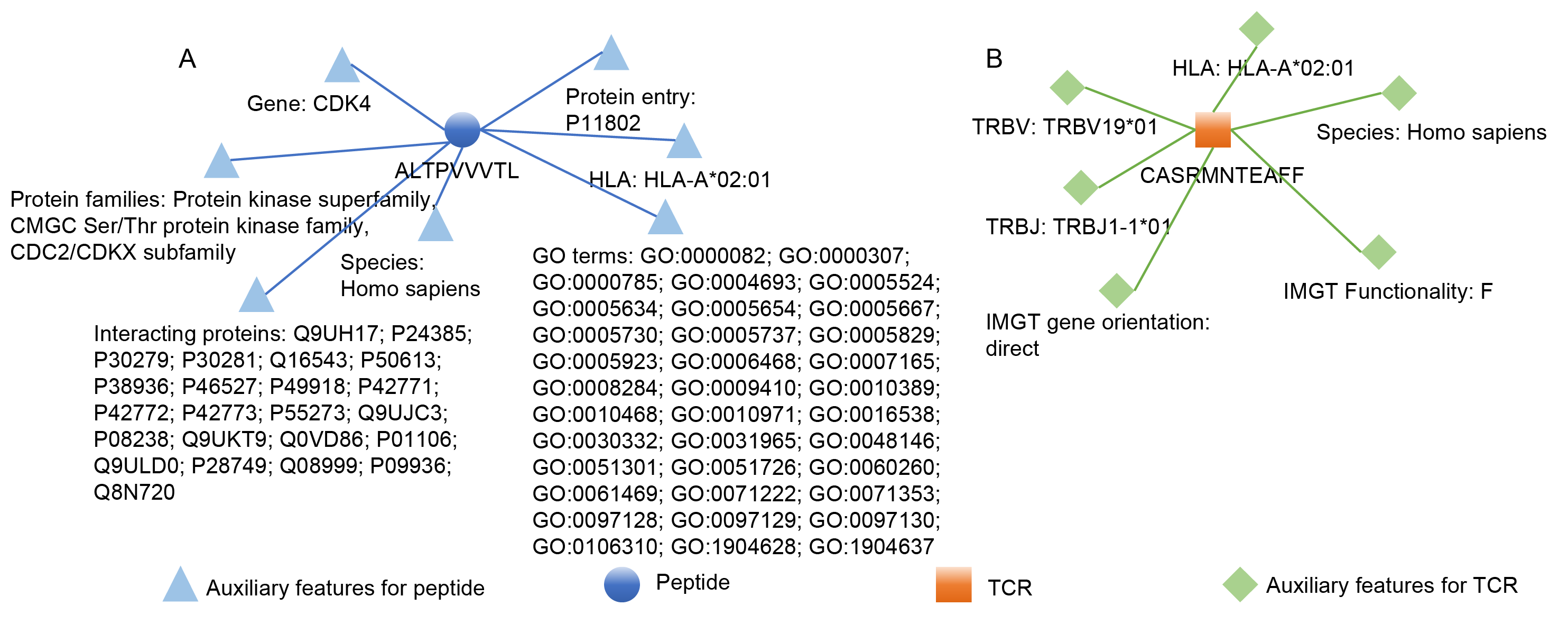
**

**Figure S4. Illustration of auxiliary features used in the construction of the CKG.**

The peptide ALTPVVVTL and the TCR sequence CASRMNTEAFF are presented as examples to demonstrate the integration of multiple biological auxiliary features.


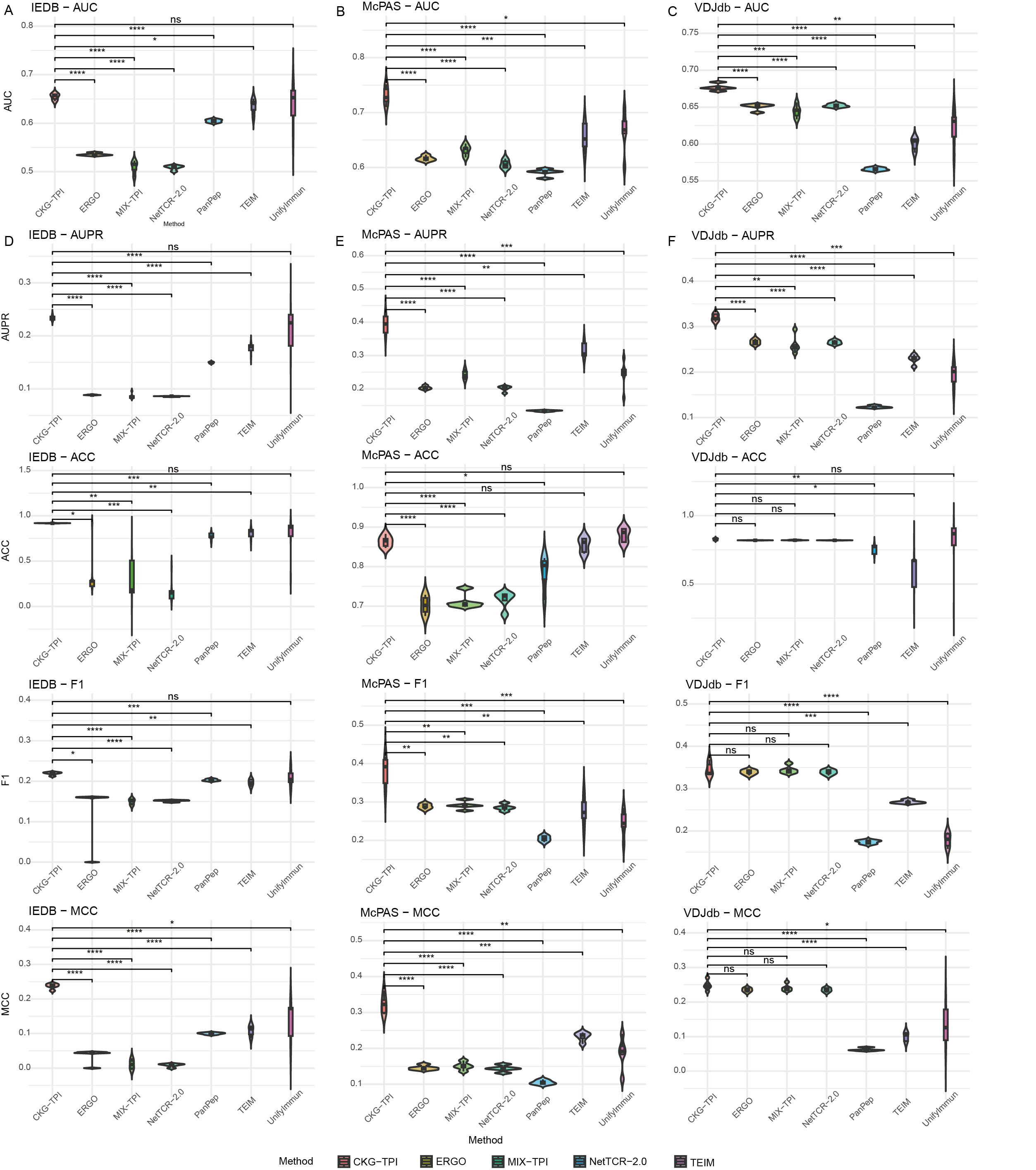


**Figure S5. Statistical significance (t-test) results of performance metrics (AUC, AUPR, ACC, F1-score, and MCC) among all methods across three independent datasets.**

"***" denotes p < 0.001, "**" denotes p < 0.01, "*" denotes p < 0.05, and "ns" denotes not significant.


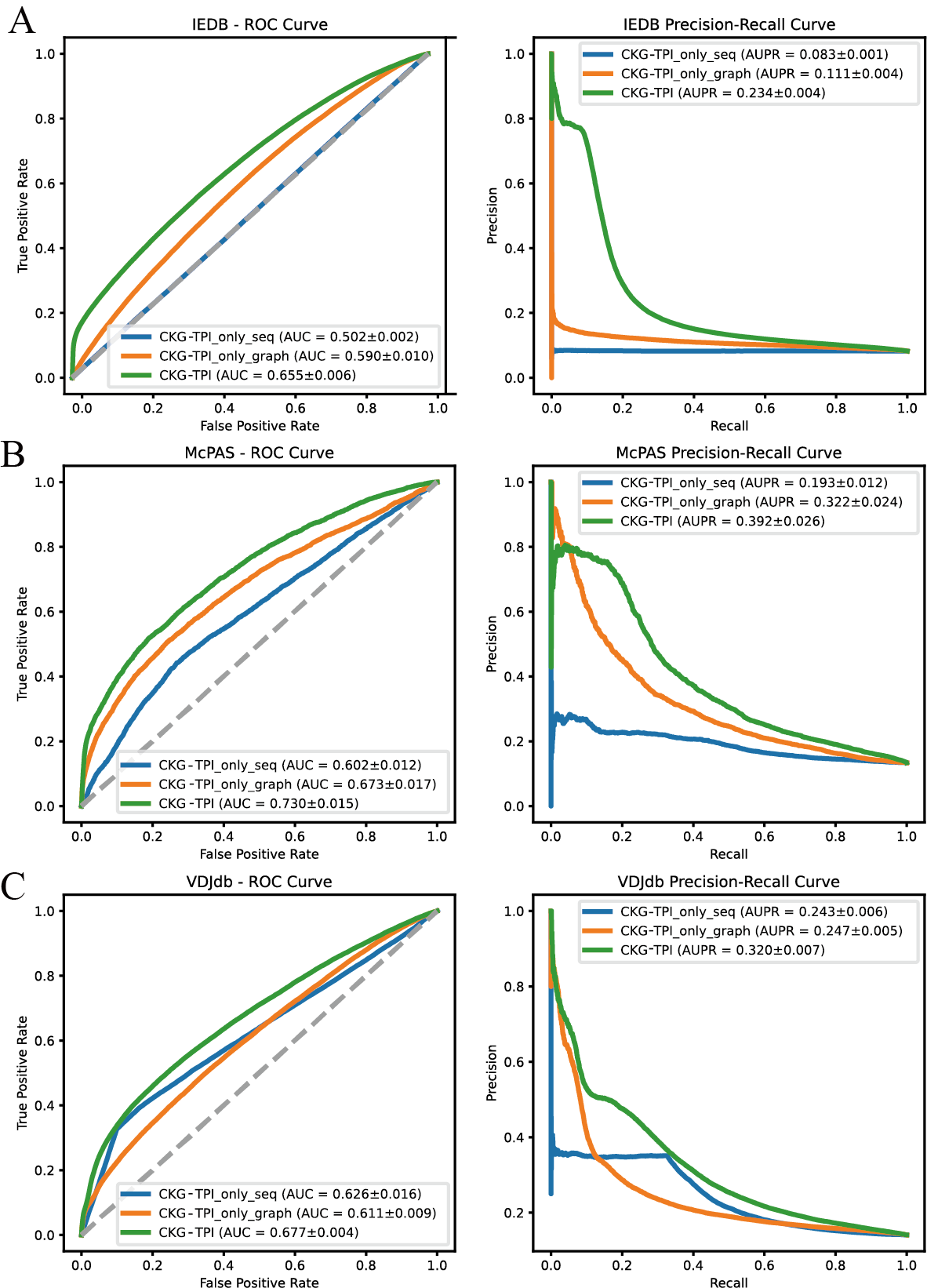


**Figure S6. Comparison of AUC and AUPR among CKG-TPI and its two variants.**

(A), (B), and (C) depict the ROC and Precision-Recall curves for the IEDB, McPAS, and VDJdb datasets, respectively. The two variants include CKG-TPI_only_graph, which retains only the knowledge graph component of CKG-TPI, and CKG-TPI_only_seq, which preserves only the sequence information component.


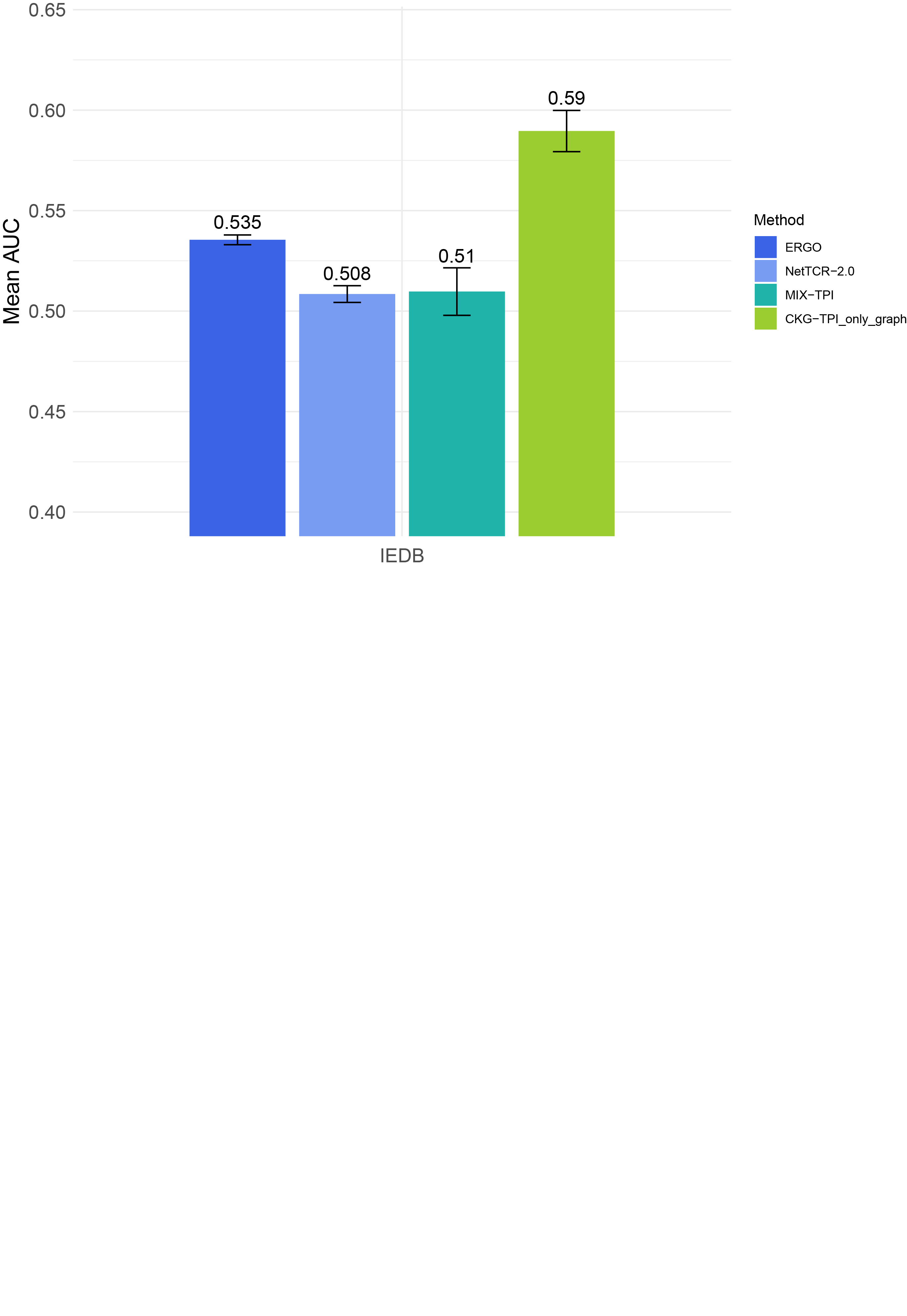


**Figure S7. AUC comparison** **between CKG-TPI_only_graph and baseline methods.**

This figure presents the mean AUC performance of **CKG-TPI_only_graph** in comparison with the baseline methods ERGO, NetTCR-2.0, and MIX-TPI.


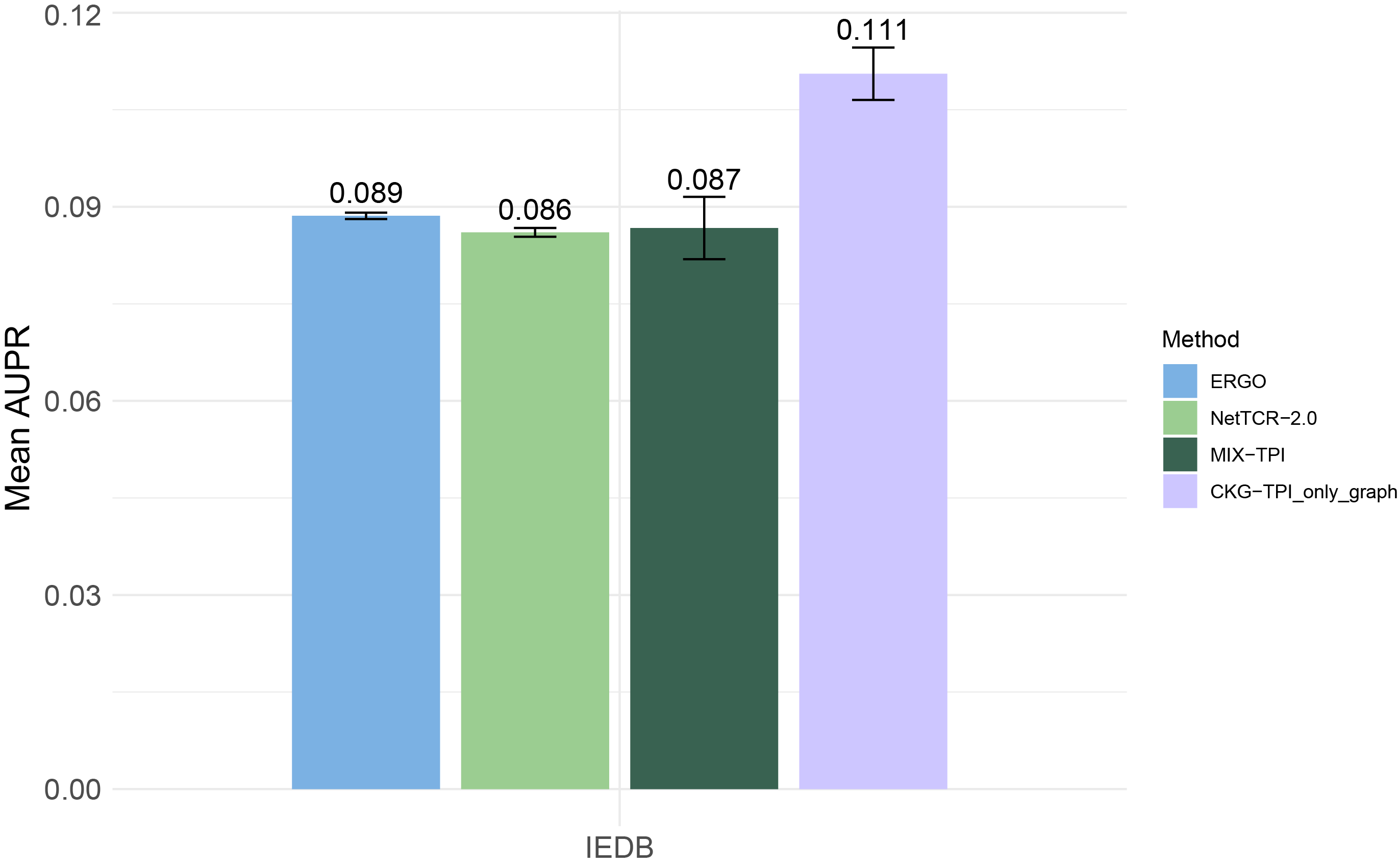


**Figure S8. Comparison of AUPR between CKG-TPI_only_graph and baseline methods.**

This figure illustrates the mean AUPR performance of CKG-TPI_only_graph, demonstrating its superiority over ERGO, NetTCR-2.0, and MIX-TPI on the IEDB dataset.


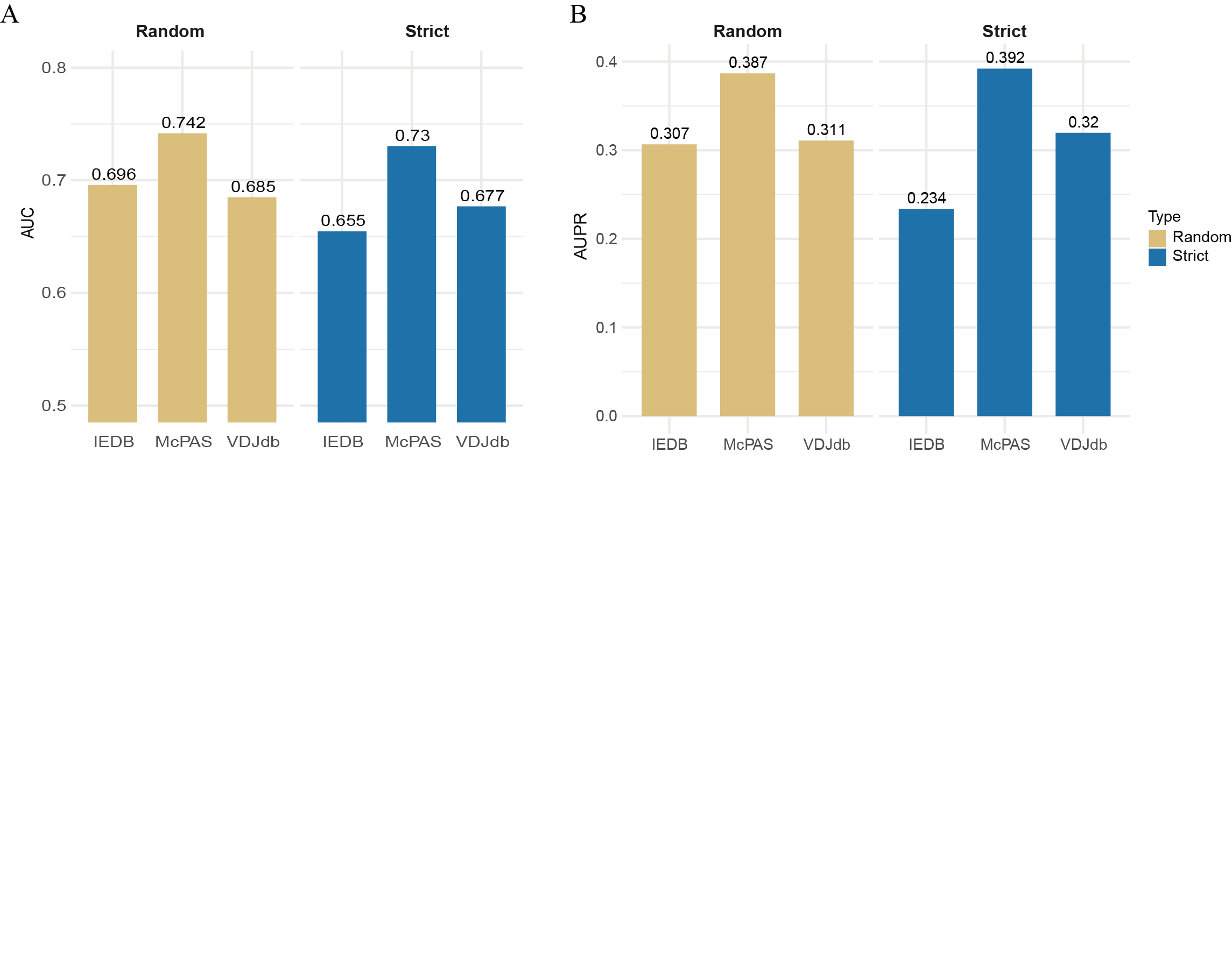


**Figure S9. Performance of CKG-TPI under two different TCR sampling strategies.**

(A) indicates the AUC performance across the three datasets, while (B) presents AUPR performance.


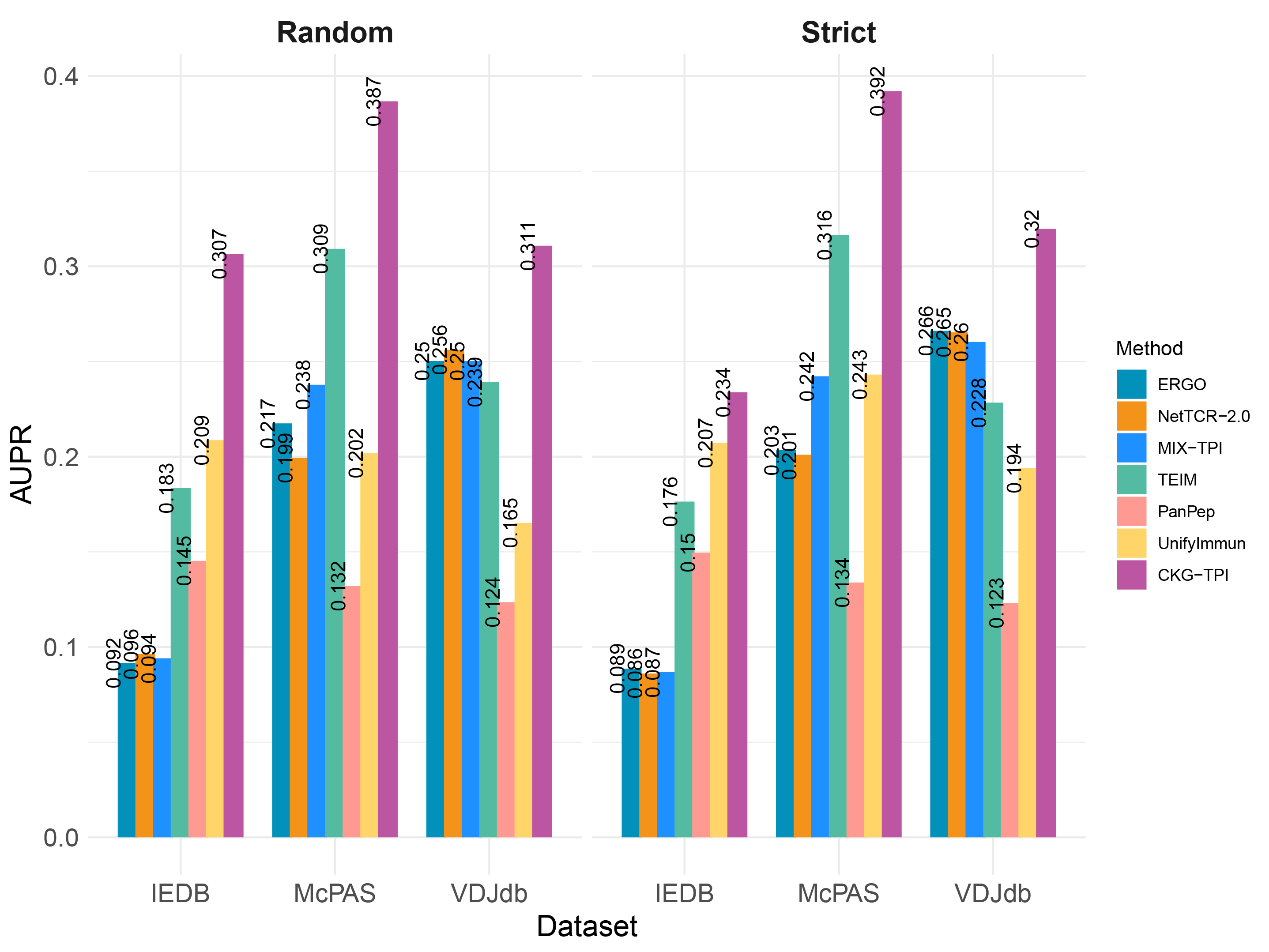


**Figure S10. AUPR performance of CKG-TPI compared to four baseline methods under two TCR sampling strategies.**

**Table S1. Parameter settings.**

| **Module_name** | **Model Parameter** | **Value** |
| --- | --- | --- |
| Embedding | Entity embedding size | 512 |
|  | Relation embedding size | 64 |
| Graph propagation module | Layers number | 3 |
|  | Output sizes for every aggregation layer | [256, 128, 64] |
|  | Message dropout for each aggregation layer | [0.2, 0.2, 0.2] |
| Interaction module | Embedding size for sequence | 256 |
|  | Transformer encoder layers number | 2 |
|  | Transformer encoder hidden size | 256 |
|  | Transformer encoder intermediate size | 1536 |
|  | Transformer encoder attention heads number | 8 |
|  | Transformer encoder attention dropout probability | 0.1 |
|  | Transformer encoder hidden dropout probability | 0.1 |
|  | Interaction module output dimension | 960 |
| Training parameters | Weight of gepre (λ) | 1e-5 |
|  | Epoch | 50 |
|  | Batch size | 128 |
|  | Learning rate | 1e-4 |

**Table S2. Performance comparison between CKG-TPI and other state-of-the-art models.**

| **Dataset** | **Method** | **Mean_AUC** | | **Mean_AUPR** | | **Mean_ACC** | **Mean_F1** | **Mean_MCC** |
| --- | --- | --- | --- | --- | --- | --- | --- | --- |
| VDJdb | ERGO | | 0.6505 | 0.2661 | 0.82 | | 0.3388 | 0.2349 |
|  | NetTCR-2.0 | | 0.6521 | 0.2653 | 0.8199 | | 0.3389 | 0.235 |
|  | MIX-TPI | | 0.6442 | 0.2602 | 0.8209 | | 0.3445 | 0.241 |
|  | TEIM | | 0.5999 | 0.2283 | 0.5927 | | 0.2685 | 0.0998 |
|  | PanPep | | 0.5659 | 0.1231 | 0.7551 | | 0.1739 | 0.0619 |
|  | UnifyImmun | | 0.6216 | 0.194 | 0.7553 | | 0.1786 | 0.1336 |
|  | CKG-TPI | | 0.6768 | 0.3196 | 0.8276 | | 0.346 | 0.2485 |
| McPAS | ERGO | | 0.6164 | 0.2034 | 0.7025 | | 0.2886 | 0.1457 |
|  | NetTCR-2.0 | | 0.606 | 0.201 | 0.7163 | | 0.2855 | 0.1438 |
|  | MIX-TPI | | 0.6317 | 0.2422 | 0.7118 | | 0.2913 | 0.1506 |
|  | TEIM | | 0.6549 | 0.3164 | 0.8548 | | 0.2763 | 0.2324 |
|  | PanPep | | 0.5911 | 0.1338 | 0.7844 | | 0.2046 | 0.1052 |
|  | UnifyImmun | | 0.6646 | 0.2431 | 0.8799 | | 0.2445 | 0.1872 |
|  | CKG-TPI | | 0.7303 | 0.3921 | 0.8634 | | 0.3788 | 0.3224 |
| IEDB | ERGO | | 0.5355 | 0.0886 | 0.3849 | | 0.1282 | 0.0359 |
|  | NetTCR-2.0 | | 0.5085 | 0.0861 | 0.1862 | | 0.1516 | 0.0083 |
|  | MIX-TPI | | 0.5097 | 0.0867 | 0.3019 | | 0.1492 | 0.0109 |
|  | TEIM | | 0.635 | 0.1764 | 0.8044 | | 0.1967 | 0.1082 |
|  | PanPep | | 0.6046 | 0.1496 | 0.7714 | | 0.2028 | 0.1001 |
|  | UnifyImmun | | 0.6367 | 0.2071 | 0.7497 | | 0.2079 | 0.1339 |
|  | CKG-TPI | | 0.6546 | 0.2338 | 0.9181 | | 0.219 | 0.2371 |

**Reference**

1. Peng, X., et al., *Characterizing the interaction conformation between T-cell receptors and epitopes with deep learning.* Nature machine intelligence, 2023. **5**(4): p. 395-407.

2. Lu, T., et al., *Deep learning-based prediction of the T cell receptor-antigen binding specificity.* Nat Mach Intell, 2021. **3**(10): p. 864-875.

3. Jiang, Y., M. Huo, and S. Cheng Li, *TEINet: a deep learning framework for prediction of TCR-epitope binding specificity.* Brief Bioinform, 2023. **24**(2).

4. Liu, H., et al., *The methods and advances of adaptive immune receptors repertoire sequencing.* Theranostics, 2021. **11**(18): p. 8945-8963.

5. Mazzotti, L., et al., *T-Cell Receptor Repertoire Sequencing and Its Applications: Focus on Infectious Diseases and Cancer.* Int J Mol Sci, 2022. **23**(15).

6. Rosati, E., et al., *Overview of methodologies for T-cell receptor repertoire analysis.* BMC Biotechnol, 2017. **17**(1): p. 61.

7. Aversa, I., et al., *Molecular T-Cell Repertoire Analysis as Source of Prognostic and Predictive Biomarkers for Checkpoint Blockade Immunotherapy.* Int J Mol Sci, 2020. **21**(7).

8. Li, N., et al., *T-cell receptor repertoire analysis for the diagnosis and treatment of solid tumor: A methodology and clinical applications.* Cancer Commun (Lond), 2020. **40**(10): p. 473-483.

9. Glanville, J., et al., *Identifying specificity groups in the T cell receptor repertoire.* Nature, 2017. **547**(7661): p. 94-98.

10. Krogsgaard, M. and M.M. Davis, *How T cells 'see' antigen.* Nat Immunol, 2005. **6**(3): p. 239-45.

11. Chronister, W.D., et al., *TCRMatch: predicting T-cell receptor specificity based on sequence similarity to previously characterized receptors.* Frontiers in immunology, 2021. **12**: p. 640725.

12. Berger, B., M.S. Waterman, and Y.W. Yu, *Levenshtein distance, sequence comparison and biological database search.* IEEE transactions on information theory, 2020. **67**(6): p. 3287-3294.

13. Giudicelli, V., D. Chaume, and M.P. Lefranc, *IMGT/GENE-DB: a comprehensive database for human and mouse immunoglobulin and T cell receptor genes.* Nucleic Acids Res, 2005. **33**(Database issue): p. D256-61.

14. Lefranc, M.P., et al., *IMGT(R), the international ImMunoGeneTics information system(R) 25 years on.* Nucleic Acids Res, 2015. **43**(Database issue): p. D413-22.

15. Korpela, D., et al., *EPIC-TRACE: predicting TCR binding to unseen epitopes using attention and contextualized embeddings.* Bioinformatics, 2023. **39**(12).

16. UniProt, C., *UniProt: the Universal Protein Knowledgebase in 2023.* Nucleic Acids Res, 2023. **51**(D1): p. D523-D531.

17. Coudert, E., et al., *Annotation of biologically relevant ligands in UniProtKB using ChEBI.* Bioinformatics, 2023. **39**(1).

18. Huang da, W., B.T. Sherman, and R.A. Lempicki, *Systematic and integrative analysis of large gene lists using DAVID bioinformatics resources.* Nat Protoc, 2009. **4**(1): p. 44-57.

19. Sherman, B.T., et al., *DAVID: a web server for functional enrichment analysis and functional annotation of gene lists (2021 update).* Nucleic Acids Res, 2022. **50**(W1): p. W216-W221.

20. Kingma, D.P., *Adam: A method for stochastic optimization.* arXiv preprint arXiv:1412.6980, 2014.
